# Three-stage vertical distribution of seawater conductivity

**DOI:** 10.1038/s41598-018-27931-y

**Published:** 2018-07-02

**Authors:** Zeyu Zheng, Yang Fu, Kaizhou Liu, Rui Xiao, Xiaohui Wang, Haibo Shi

**Affiliations:** 1 Shenyang Institute of Automation, Chinese Academy of Sciences, Shenyang, 110016 PR China; 2 Key Laboratory of Network Control System, Chinese Academy of Sciences, Shenyang, 110016 PR China; 3 State Key Laboratory of Robotics, Chinese Academy of Sciences, Shenyang, 110016 PR China; 4 Department of Biostatistics and Epidemiology, University of Pennsylvania Perelman School of Medicine, Philadelphia, PA 19104 USA; 5 University of Chinese Academy of Sciences, Beijing, 100049 China

## Abstract

Seawater conductivity is an important indicator of ocean electromagnetic properties and directly impacts the electromagnetic attenuation characteristics and phase distribution features of the ocean. Few studies have considered how the combined effects of salinity, temperature and pressure affect the vertical conductivity distribution and its formation mechanisms. Here, we analyse the vertical distributions of seawater conductivity from the sea surface to a maximum depth of 7062 m at five different locations. Electric conductivity profiles show similar vertical structures at all locations. Electric conductivity decreases with increasing depth first and then slowly increases from approximately 2000 m to the seabed. We observe an exponential relationship between the conductivity minimum and the water depth. At all five measurement locations, seawater conductivity measurements show a stable three-stage vertical distribution on logarithmic scales, with the middle stage satisfying a power law relationship. We analyse the vertical distribution of temperature in the second stage and investigate the relationship between temperature and conductivity. The results show that temperature also exhibits a power-law relationship with depth and a high linear correlation exists between temperature and conductivity. Our findings suggest that the vertical structure of conductivity is largely temperature dependent.

## Introduction

Seawater conductivity refers to the conductive capability of seawater and can help describe the electromagnetic properties of the ocean^1,2^. As interest in physical oceanography increases, the distribution and variability of seawater conductivity have become important research topics^3–5^. Previous studies have revealed that changes in seawater conductivity strongly impact the electromagnetic attenuation characteristics and phase distribution features of the seawater, which directly affect communication and navigation in the ocean^6,7^. Moreover, seawater conductivity has also been widely used to measure and study sea salinity^8–10^.

Current theory suggests that the vertical distribution of seawater conductivity is associated with salinity, temperature and pressure^11,12^. It is generally accepted that seawater conductivity is proportional to seawater salinity^13,14^, and previous studies have shown that seawater temperature strongly influences seawater conductivity^15,16^. Furthermore, pressure affects seawater conductivity by changing the seawater density and thus changing the concentration of ions in seawater^17,18^. Pressure also affects the chemical equilibrium of compounds in seawater (e.g., magnesium sulfate), which may shift reactions in different directions and alter the ionic composition of seawater^19^. For example, it is reported that the dissociation of MgSO_4_ is approximately twice as effective at 2000 bars than at 1 bar^20^. However, few studies have focused on the combined effects of salinity, temperature and pressure and how they affect seawater conductivity or the formation mechanisms of vertical conductivity distribution^21,22^. Recent studies have concentrated on the effects of seawater conductivity on electromagnetic induction in the ocean^23,24^, which requires accurate measurements of seawater conductivity and can potentially be used to indirectly observe the ocean and its interior^25^. For instance, Irrgang *et al*. (2015) examined the spatial and temporal influence of variable seawater conductivity on motional induction simulated with an ocean general circulation model and revealed that using instead a realistic global seawater conductivity distribution increases the temporal variability of the magnetic field up to 45%^26^. Thus, a comprehensive understanding of the seawater conductivity distribution is essential.

Here, we use the seawater conductivity measurements collected by the manned submersible, Jiaolong^27–29^, to investigate the vertical structure of seawater conductivity from the surface to the seabed and explore the formative mechanisms of the vertical conductivity distribution. Conductivity was measured in the South China Sea, the waters near the Republic of Kiribati, the sea near the Mariana Trench, the western Pacific Ocean and the Southwestern Indian Ocean. We find that the conductivity observations all exhibit a similar three-stage vertical distribution and show a gradual increase in the deep sea. The minimum value of conductivity and its corresponding depth are both exponential functions of the total ocean depth. The conductivity measurements show a three-stage vertical distribution across our study sites, and a power law best describes the observed relationship in the middle stage. Furthermore, as the conductivity measurements were only collected from the mid-low latitudes of the Pacific and Indian, we used the Atlantic CTD data archived in World Ocean Atlas to make a comparative analysis of the vertical conductivity structures in different areas.

## Results

### Vertical distribution of deep-sea conductivity

**Figure 1:**
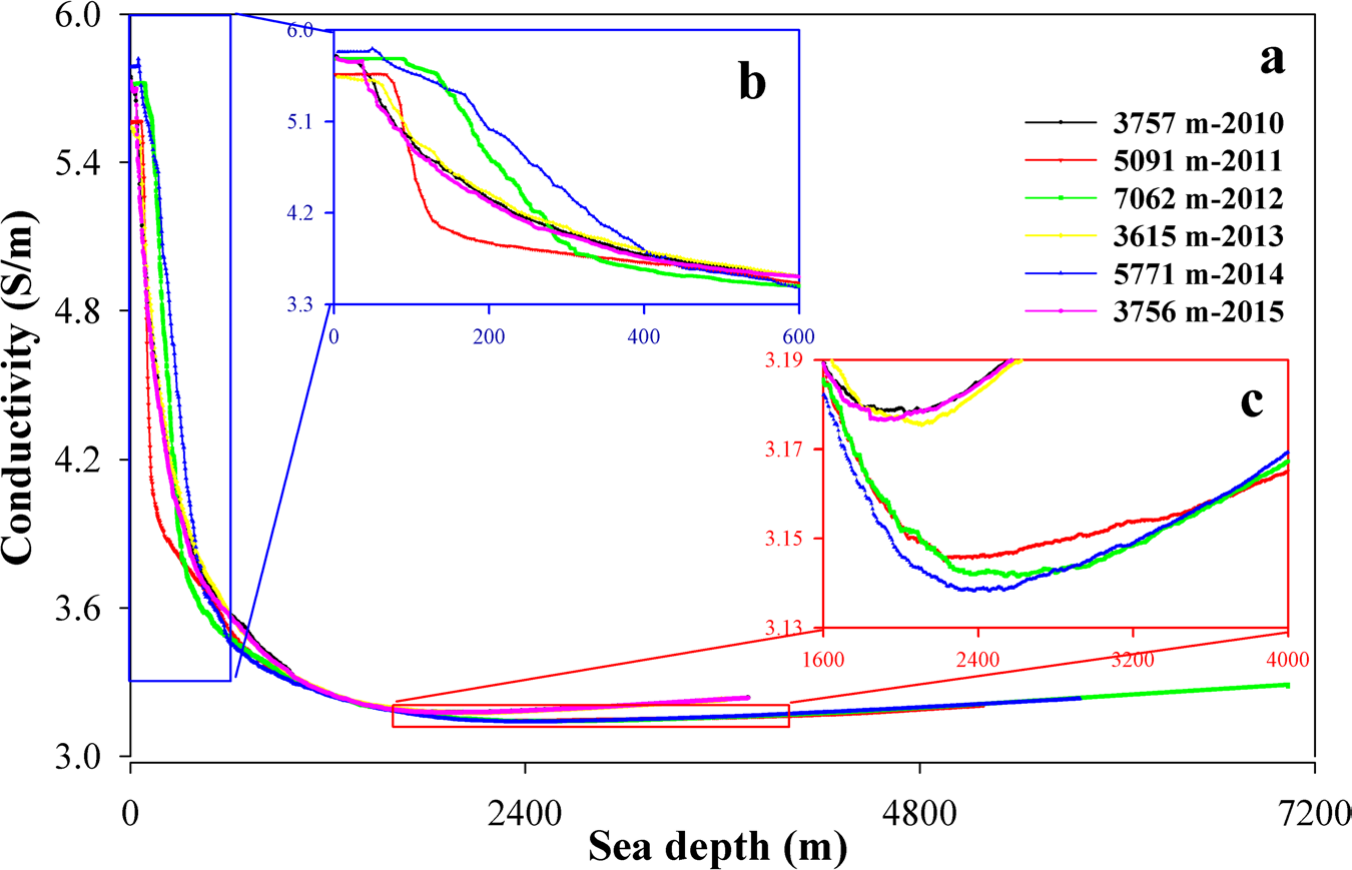
The vertical conductivity measured at five representative locations. (**b**) and (**c**) are the magnified red and blue boxes, respectively, in (**a**). The x and y-axes are exaggerated to accentuate differences.

Measurements from surface to 3000, 5000 and 7000 m depths, conducted each year from 2010–2015, show an exponential decrease in conductivity with depth (Fig. 1a). The seawater conductivity at all locations ranges between 3 and 6 S/m. The maximum values of conductivity occur in the shallow water between 20 and 100 m below the surface, while the minimum values of conductivity occur in the middle water between 1800 and 2600 m. Focusing on the depth intervals 0–600 m and 1600–4000 m facilitates visualization of our results. For instance, seawater conductivity initially increases with depth from the surface and then decreases rapidly between 80 and 400 m (Fig. 1b). The seawater conductivities increase slowly with depth again at a depth from approximately 2000 m to the sea-bed (Fig. 1c). Seawater conductivity shows a similar rapidly decreasing trend between 80 and 400 m in the 3000 m depth measurements from 2010, 2013 and 2015 and in the 5000 and 7000 m depth measurements. Moreover, the position of the conductivity minimum varies among the three depth class measurements.

### Stable three-stage vertical distributions in logarithmic conductivity-depth relationship

**Figure 2:**
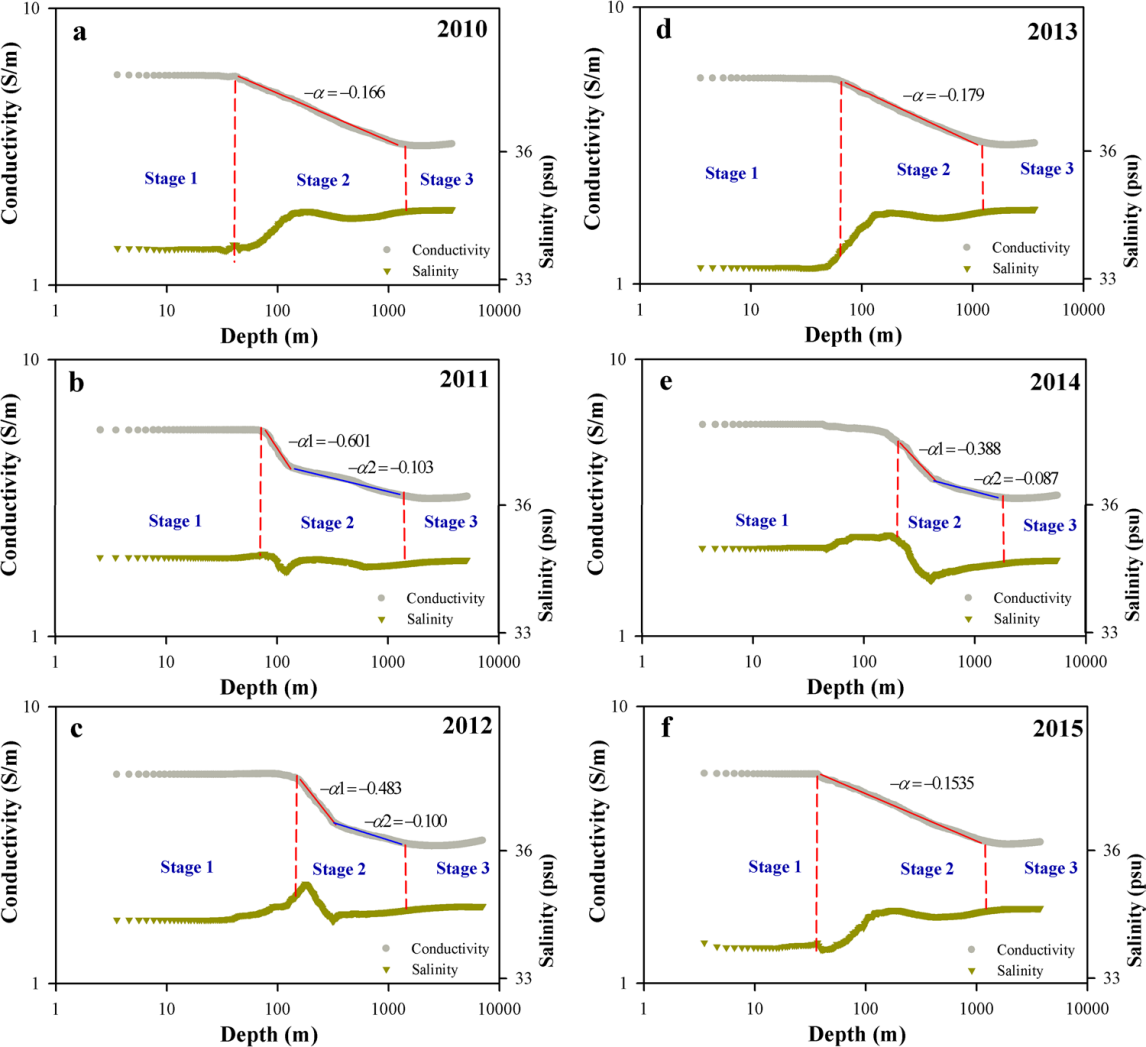
Log–log plot of the relationships between conductivity and water depth and between salinity and water depth. The single power exponent, α, is indicated by the line segment in red. The two exponents are indicated by the two red and blue line segments. The coefficients of determination (R^2^) values for the line segments range from 0.991 to 0.998.

The relation between water depth and conductivity is presented on a logarithmic scale in Fig. 2, in which three apparent stages can be seen. In the first stage, conductivity is relatively constant, as measurements show little variation. In the second stage, water depth and conductivity show a linear relationship on logarithmic scale that can be described by the power law.

1 $$C(d)\sim {d}^{-\alpha }$$

where *C*(*d*) is the electrical conductivity of seawater, *d* is water depth and *α* is the power exponent.

The connections between water depth and conductivity differ among the depth class measurements. For instance, this relationship can be described by a single power law exponent, *α*, in the 3000 m depth class measurements from 2010, 2013 and 2015, while the measurements in the 5000 and 7000 m depth classes are better fit by two power law exponents, *α*1 and *α*2. The two red and blue line segments indicate *α*1 and *α*2, respectively, and the coefficients of determination (R^2^) for the line segments range from 0.991 to 0.998, respectively. As for the third stage, seawater conductivity has minor fluctuations and the magnitude of conductivity change is small on logarithmic scale.

Most recent studies have focused on seawater salinity rather than electric conductivity^30–32^. To investigate the differences between conductivity and salinity, we compared the seawater conductivity-depth relationship with the salinity-depth relationship on a log-log plot. The results indicated that the vertical distribution of conductivity is less affected by geographic location than salinity. In contrast, the vertical distribution of salinity is dependent on geographical locations. For instance, the vertical distribution of salinity is significantly different between the Tropical Ocean and the Temperate Ocean, yet seawater conductivity remains unchanged (Fig. 2a,b).

### Depth ranges of the three stages within the vertical conductivity distribution

**Figure 3:**
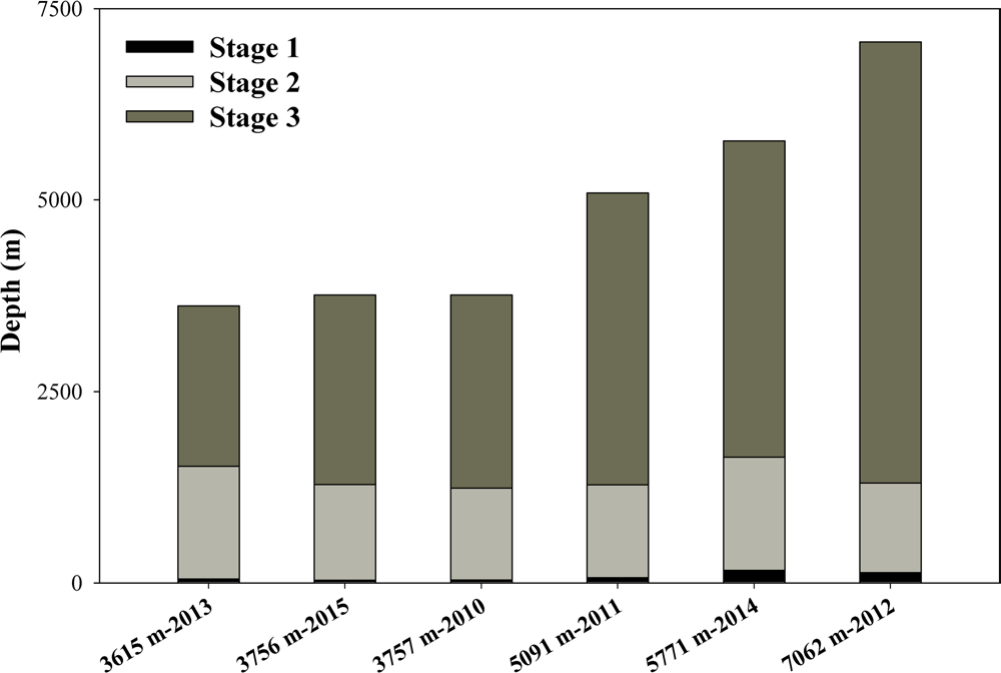
The depth ranges of the three stages within the vertical conductivity distribution.

By analysing the depth ranges of the three stages within the vertical conductivity distribution, we find that the first stage ranges from the sea surface to 166 m and constitutes approximately 1% of the total depth profile. The second stage ranges from 16%–40% of the total depth and transitions into the third stage, which spans 58%–82% of the vertical profile, between 1300–1600 m. The proportions of the second and third stages increase concurrently with total water depth (Fig. 3).

### Influence of the total water depth on the depth of minimum seawater conductivity

**Figure 4:**
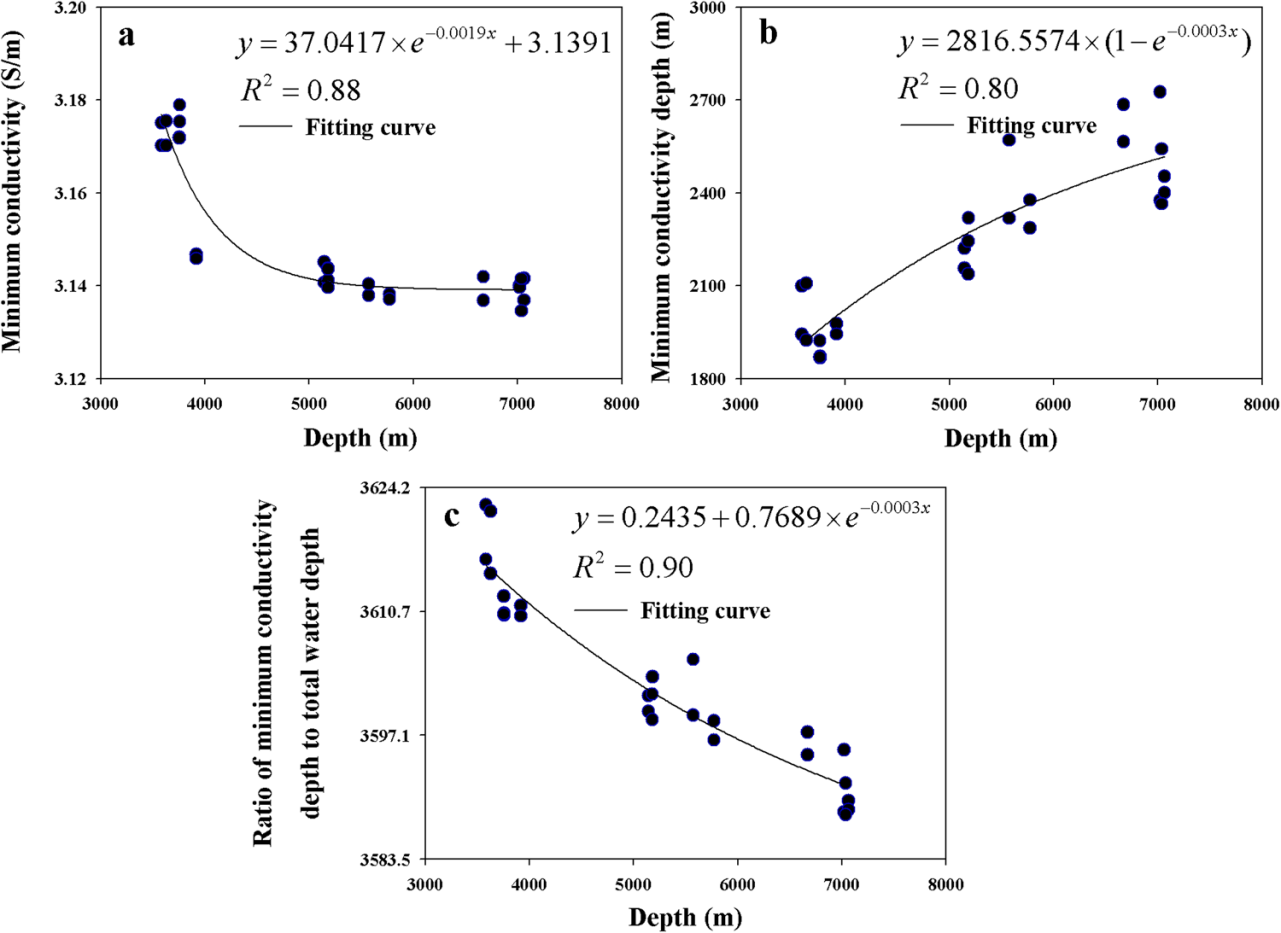
Relationships between the conductivity minimum and total water depth. (**a**) Minimum seawater conductivity vs. total water depth. (**b**) Minimum conductivity depth vs. total water depth. (**c**) Total water depth vs. the ratio of minimum conductivity depth to total water depth. The solid line is the fitting line.

The seawater conductivity measurements at each location show a gradual increase with depth from approximately 2000 m to the seabed. We find that the relationship between minimum seawater conductivity and total water depth can be described by an exponential relationship ($y=37.0417\times {e}^{-0.0019x}+3.1391$) with an R^2^ value of 0.88 (Fig. 4a). A similar relationship exists between the depth of minimum seawater conductivity and total water depth ($y=2816.5574\times (1-{e}^{-0.0003x})$) with an R^2^ value of 0.80 (Fig. 4b). Additionally, the relationship between ratio of the depth of minimum seawater conductivity to the total water depth and the total water depth can also be described by an exponential relationship ($y=0.2435+0.7689\times {e}^{-0.0003x}$) with a high R^2^ value of 0.90 (Fig. 4c). From these relationships, we can conclude that the depth of minimum seawater conductivity increases with total water depth. For example, the average depths of minimum seawater conductivity are approximately 1900, 2300 and 2600 m in the 3000, 5000 and 7000 m depth class measurements, respectively.

## Discussion

The results shown about are based on conductivity measurements collected from the mid-low latitudes of the Pacific and Indian Oceans. Local variations in salinity and temperature may cause differences in the vertical conductivity structure in distinct geographic locations^33^. For example, previous research has reported decreases in Atlantic Ocean water salinity with depth, which is opposite of the observed trend in the Pacific^34^. Therefore, we compared the vertical conductivity structures in the Pacific and the Indian Ocean with that of the Atlantic ocean based on three CTD profiles from the World Ocean Database 2013 (WOD13). The results indicate that the vertical conductivity structures in the Atlantic are similar with the ones which observed in the Pacific and the Indian Ocean (Fig. S1). The relation between conductivity and water depth on a logarithmic scale can still be divided into three apparent stages and those conductivity profile show consistent power-law relationships with depth in the second stage.

This study investigated the vertical structure of conductivity at different seawater depths and provides measurements of seawater conductivity from the surface to a maximum depth of 7062 m. Generally, seawater conductivity decreases exponentially with depth. By displaying the data on a log-log plot, three distinct relationships (stages) between water depth and conductivity were evident along the vertical water column. The first stage is approximately 1% of the total water depth and occurs several decametres deep. Within the first stage, there is little variation in conductivity, and the relationship between depth and conductivity can be approximated by a constant on the log-log axes. The little observed variation in conductivity can be attributed to the well-mixed and relatively uniform characteristics of the oceanic surface mixed layer^35^.

**Figure 5:**
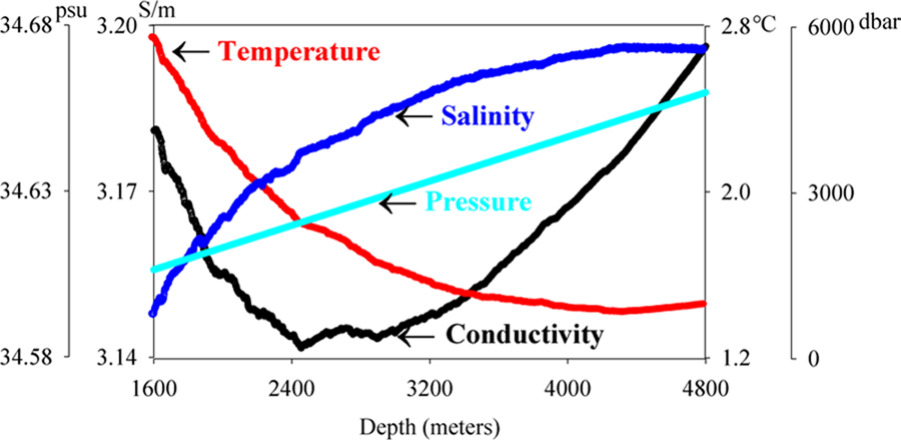
The vertical distribution characteristics of deep-sea conductivity and the relevant controls.

In the third stage, seawater conductivity exhibited slow rise with depth. We attributed the increase of conductivity with depth in the deep ocean to three reasons. First, previous studies have indicated that deep sea temperatures slowly rise due to adiabatic heating^36^, leading to an increase in conductivity values. Second, high seawater pressure at great depths can increase the concentration of ions in seawater, which also leads to an increase in conductivity. Third, we hypothesize that mineral salt from the seabed dissolves in seawater, diffusing upward until the salinity reaches upper ocean levels^37^. This phenomenon would also explain our increasing measurements of conductivity with depth in the deep ocean. Additionally, we use 2012 data at depths from 1600–4800 m to characterize the vertical distribution of deep-sea conductivity and to analyse the potential controls on this feature (Fig. 5). The results indicate that both seawater salinity and pressure increase while seawater temperature decreases with depth in the vicinity of the minimum seawater conductivity. Increasing salinity and pressure can increase the seawater conductivity, while decreasing the temperature has the opposite effect^38,39^. Thus, the observed increases in deep ocean conductivity and the location of the minimum seawater conductivity can be attributed to the combined effects of temperature, pressure, and salinity. Moreover, we note that the depth of the conductivity minimum and the relative proportion of the third stage in the water column increase concurrently with total water depth. This phenomenon is related to the increasing salinity and pressure in the third stage. As pressure increases linearly, the most likely reason for this is the upward dissolution of salt in the seabed minerals and rocks, and it is regulated by vertical convection and thermohaline circulation^40,41^.

**Figure 6:**
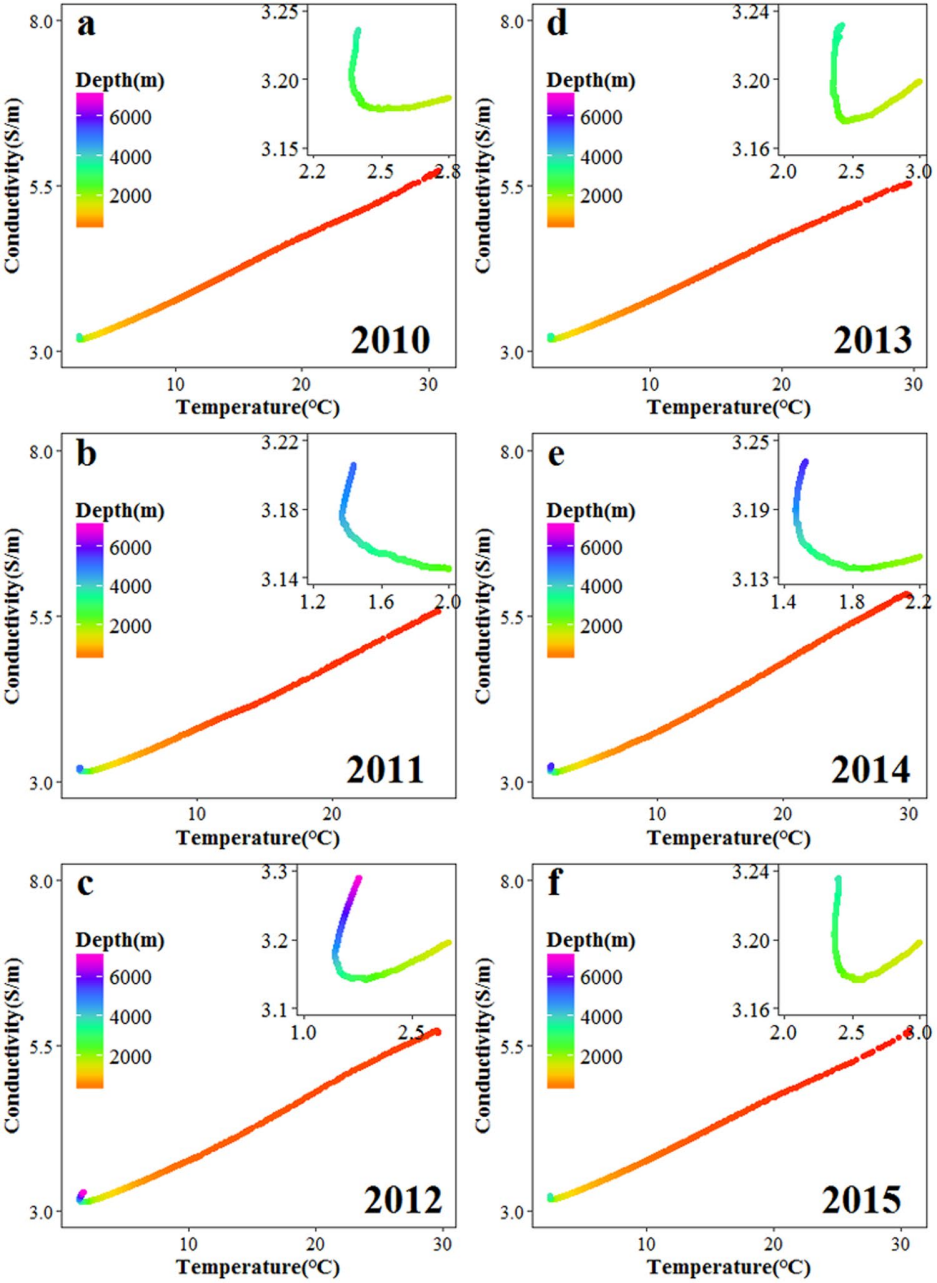
Relationship between temperature and conductivity. The inset panels are the partial enlarged display of the relationship between temperature and conductivity in the deep water.

Previous studies have not detailed the conductivity distribution and formative mechanisms of the middle layer water^42^. In this study, the observed relationship between conductivity and water depth in the middle layer exhibited a power-law relationship. It is usually considered that the vertical distribution of conductivity is mainly affected by the combined action of temperature, pressure, and salinity, which has been discussed in this paper. Our results have shown that conductivity decreases with depth in both the Pacific and Atlantic. Since temperature is the only one of those three variables that decreases with depth in both oceans, this may suggest that the conductivity in the Pacific and Atlantic is, to the first order, regulated by temperature. Therefore, we analyzed the vertical distribution of temperature in the second stage and investigated the relationship between temperature and between conductivity. The results show that temperature also reveals a power-law relationship with depth in the second stage (Fig. S2) and a high linear correlation exists between temperature and conductivity from sea surface to 2000 m depth (Fig. 6). By correlation analysis, all the correlation coefficients between temperature and conductivity range from 0.98 to 0.99 (Table S1). Our findings supported the idea that the vertical structure of conductivity is largely temperature dependent.

## Data and Methods

**Figure 7:**
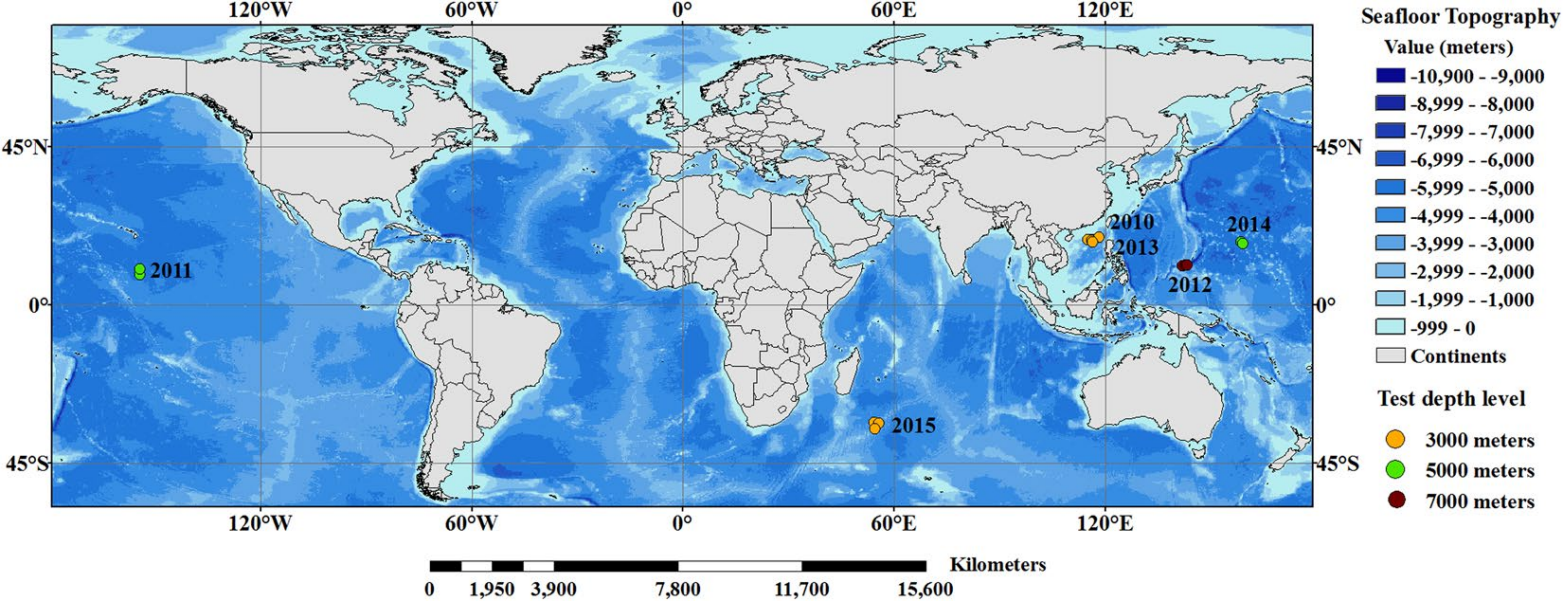
Map showing the locations of the study sites. The seafloor topography is based on the ETOPO1 bathymetric grid data (https://data.nodc.noaa.gov/cgi-bin/iso?id = gov.noaa.ngdc.mgg.dem:316)^50^. This map is generated using ArcGIS (version 9.3) created by the ESRI Corporation (https://www.esri.com/zh-cn/home)^51^.

Conductivity in this investigation was measured by the conductivity-temperature-depth (CTD) on board the Jiaolong manned submersible from 2010 to 2015^43,44^. During this period, the 3000, 5000 and 7000 m depth measurements were collected. The study sites were located in the South China Sea, the waters near the Republic of Kiribati, the sea near the Mariana Trench, the western Pacific Ocean and the Southwestern Indian Ocean (Fig. 7). Detailed information on data collection at each of these sites is provided in Table 1. These data were measured closely following the rigorous, community-defined experiment deployment and data collection protocols^45^. During the analysis of the vertical conductivity structure, we selected the average conductivity of each depth class to represent the vertical conductivity structure. During the analysis of the minimum conductivity values, we thoroughly searched through each dataset for the conductivity minimum and its depth position. Besides that, three CTD profiles were taken from the World Ocean Database 2013 (WOD13), provided by the National Oceanographic Data Center (NODC), to test whether the differences in the vertical conductivity structures from different areas are existent (Table S2). Since there is no conductivity data in the WOD13 data set, conductivity was calculated by salinity, temperature and pressure using the Practical Salinity Scale 1978^46,47^.

**Table 1:** Detailed information of Jiaolong manned submersible data collection.

Year	Measurement depth (m)	Measurement repetitions	Date	Latitude	Location
2010	3000	1	12th July	low latitudes	South China Sea Seamounts
2011	5000	3	27th July, 29th July and 31st July	low latitudes	Sea near the Republic of Kiribati
2012	7000	4	15th June, 24th June, 27th June, and 30th June	low latitudes	Micronesia exclusive economic zone in the southern Mariana Trench
2013	3000	4	28th June, 3rd July, 5th July and 7th July,	low latitudes	South China Sea Seamounts
2014	5000	2	19th July and 27th July	low latitudes	Western Pacific
2015	3000	3	10th January, 2nd February and 9th July	middle latitudes	Southwestern Indian Ocean

The Practical Salinity Scale 1978 is based on an equation relating salinity to the ratio of the electrical conductivity of seawater at 15 °C to that of a standard potassium chloride solution (KCl)^48^. According to the algorithm of the PSS 78, the ratio (***R***) of the *in-situ* electrical conductivity of seawater of practical salinity ***S***, at temperature ***T*** and pressure ***P*** to that of seawater of practical salinity 35‰ at 15 °C and 0 dbar can be expressed as follows:

2 $$R=\frac{C(S,T,P)}{C(35,15,0)}$$

Here, ***C***(***S***, ***T***, ***P***) is the *in-situ* electrical conductivity of seawater of practical salinity ***S***, at temperature ***T*** and pressure ***P***. ***C***(35, 15, 0) is the conductivity of standard seawater (35‰) at 15 °C which by definition has a conductivity equal to that of the standard KCl solution at that temperature and its value is 4.2914 Siemens/meter^49^. Temperature is in degrees Celsius (IPTS-68) and the units of pressure are decibars in accordance with oceanographic practice.

In order to simplify the data reduction process, the conductivity ratio ***R*** is usually separated into three factors ***R***_***p***_, ***r***_***T***_, and ***R***_***p***_, which can be expressed as follows:

3 $${R}_{p}=\frac{C(S,T,P)}{C(S,T,0)}$$

4 $${r}_{T}=\frac{C(35,T,0)}{C(35,15,0)}$$

5 $${R}_{T}=\frac{C(S,T,0)}{C(35,T,0)}$$

6 $$R={R}_{p}{R}_{T}{r}_{T}$$

Here, ***r***_***T***_ and ***R***_***p***_ are given empirical functions as follows:

7 $${r}_{T}={c}_{0}+{c}_{1}T+{c}_{2}{T}^{2}+{c}_{3}{T}^{3}+{c}_{4}{T}^{4}$$

$$\begin{array}{rcl}{c}_{0} & = & 6.766097\times {10}^{-1}\\ {c}_{1} & = & 2.00564\times {10}^{-2}\\ {c}_{2} & = & 1.104259\times {10}^{-4}\\ {c}_{3} & = & -6.9698\times {10}^{-7}\\ {c}_{4} & = & 1.0031\times {10}^{-9}\end{array}$$

8 $${R}_{p}=1+\frac{{A}_{1}P+{A}_{2}{P}^{2}+{A}_{3}{P}^{3}}{1+{B}_{{\rm{1}}}T+{B}_{{\rm{2}}}{T}^{2}+{B}_{{\rm{3}}}{T}^{3}+{B}_{{\rm{4}}}TR}$$

$$\begin{array}{rcl}{A}_{1} & = & 2.070\times {10}^{-5}\\ {A}_{2} & = & -6.370\times {10}^{-\mathrm{10}}\\ {A}_{3} & = & 3.989\times {10}^{-\mathrm{15}}\\ {B}_{{\rm{1}}} & = & 3.426\times {10}^{-2}\\ {B}_{{\rm{2}}} & = & 4.464\times {10}^{-4}\\ {B}_{{\rm{3}}} & = & 4.215\times {10}^{-1}\\ {B}_{{\rm{4}}} & = & -3.107\times {10}^{-3}\end{array}$$

And salinity ***S***, ***R***_***T***_ and ***T*** satisfy the following polynomials:

9 $$\begin{array}{c}S={a}_{0}+{a}_{1}{{R}_{T}}^{1/2}+{a}_{2}{R}_{T}+{a}_{3}{{R}_{T}}^{3/2}+{a}_{4}{{R}_{T}}^{2}+{a}_{5}{R}_{T}\\ +\frac{T-15}{1+K(T-15)}[{b}_{0}+{b}_{1}{{R}_{T}}^{1/2}+{b}_{2}{R}_{T}+{b}_{3}{{R}_{T}}^{3/2}+{b}_{4}{{R}_{T}}^{2}+{b}_{5}{{R}_{T}}^{5/2}]\end{array}$$

$$\begin{array}{rcl}{a}_{0} & = & 8\times {10}^{-3}\\ {a}_{1} & = & -1.692\times {10}^{-1}\\ {a}_{2} & = & 25.3851\\ {a}_{3} & = & 14.0941\\ {a}_{4} & = & -7.0261\\ {a}_{5} & = & 2.7081\\ {b}_{0} & = & 5\times {10}^{-4}\\ {b}_{1} & = & -5.6\times {10}^{-3}\\ {b}_{2} & = & -6.6\times {10}^{-3}\\ {b}_{3} & = & -3.75\times {10}^{-2}\\ {b}_{4} & = & 6.36\times {10}^{-2}\\ {b}_{5} & = & -1.44\times {10}^{-2}\\ K & = & 1.62\times {10}^{-2}\end{array}$$

Thus the *in-situ* electrical conductivity can be computed using equation (2), (6), (7), (8), (9) and the existing salinity, temperature and pressure data.

## Data availability

The conductivity-temperature-depth (CTD) profiles from the Jiaolong manned submersible were provided under license from the Ocean Mineral Resource Research and Development Association of China and so are not publicly available. They are available from the authors upon reasonable request and with permission from the Ocean Mineral Resource Research and Development Association of China. Additionally, three CTD profiles were taken from the World Ocean Database 2013 (WOD13), provided by the National Oceanographic Data Center (NODC). These three CTD profiles data are available at https://www.nodc.noaa.gov/OC5/SELECT/dbsearch/dbsearch.html. The seafloor topography was based on the ETOPO1 bathymetric grid data. The ETOPO1 bathymetric grid data are available at https://data.nodc.noaa.gov/cgi-bin/iso?id=gov.noaa.ngdc.mgg.dem:316.

## Electronic supplementary material

Supporting Information
