# Supplementary material for: Three-stage vertical distribution of seawater conductivity
Source: Sci Rep. 2018 Jul 2;8:9916. doi: 10.1038/s41598-018-27931-y (PMC6028440; doi:10.1038/s41598-018-27931-y)
Supplement: Supplementary file 1 — Supporting Information [file 41598_2018_27931_MOESM1_ESM.pdf]

Scientific Reports

Supporting Information for

Three-stage vertical distribution of seawater conductivity

Zeyu Zheng[1, 2#*], Yang Fu[1, 2#], Kaizhou Liu[1, 3], Rui Xiao[4], Xiaohui Wang[1, 3], Haibo Shi[1, 2]

[1]Shenyang Institute of Automation, Chinese Academy of Sciences, Shenyang, 110016, PR China

[2]Key Laboratory of Network Control System, Chinese Academy of Sciences, Shenyang, 110016, PR China

[3]State Key Laboratory of Robotics, Chinese Academy of Sciences, Shenyang, 110016, PR China

[4]Department of Biostatistics and Epidemiology, University of Pennsylvania Perelman School of Medicine, Philadelphia, PA 19104, USA

Contents of this file

Figure S1. The vertical conductivity measured in the mid-low latitudes of the Atlantic. The inset panels show the relationships between conductivity and water depth on logarithmic scale. The single power exponent, α, is indicated by the line segment in red. The coefficients of determination (R^2) values for the line segments range from 0.98 to 0.99.

Figure S2. Log–log plot of the relationship between temperature and water depth

[*]Author for correspondence: Zeyu Zheng: zhengzeyu@sia.cn
[#]These authors contributed equally to this work.

at five representative locations. The short dash segment is the fitting line. The coefficients of determination (R^2) values for the short dash segments range from 0.91 to 0.99.

Table S1. Correlation analysis between temperature and conductivity.

Table S2. Detailed information of three CTD profiles in the Atlantic.

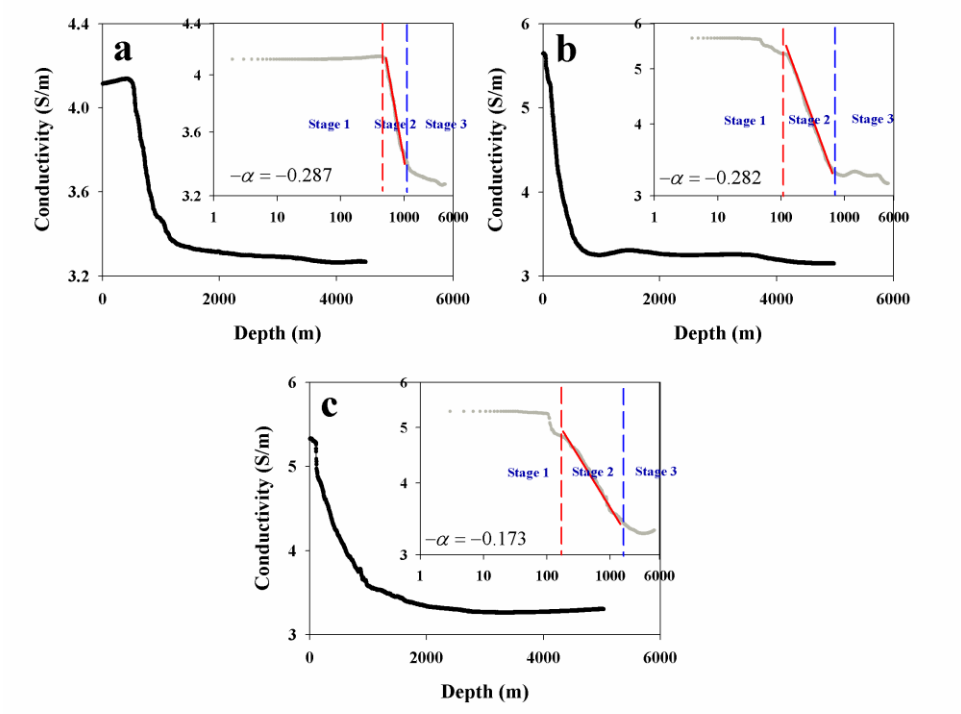

Figure S1. The vertical conductivity measured in the mid-low latitudes of the Atlantic. The inset panels show the relationships between conductivity and water depth on logarithmic scale. The single power exponent, α, is indicated by the line segment in red. The coefficients of determination (R^2) values for the line segments range from 0.98 to 0.99.

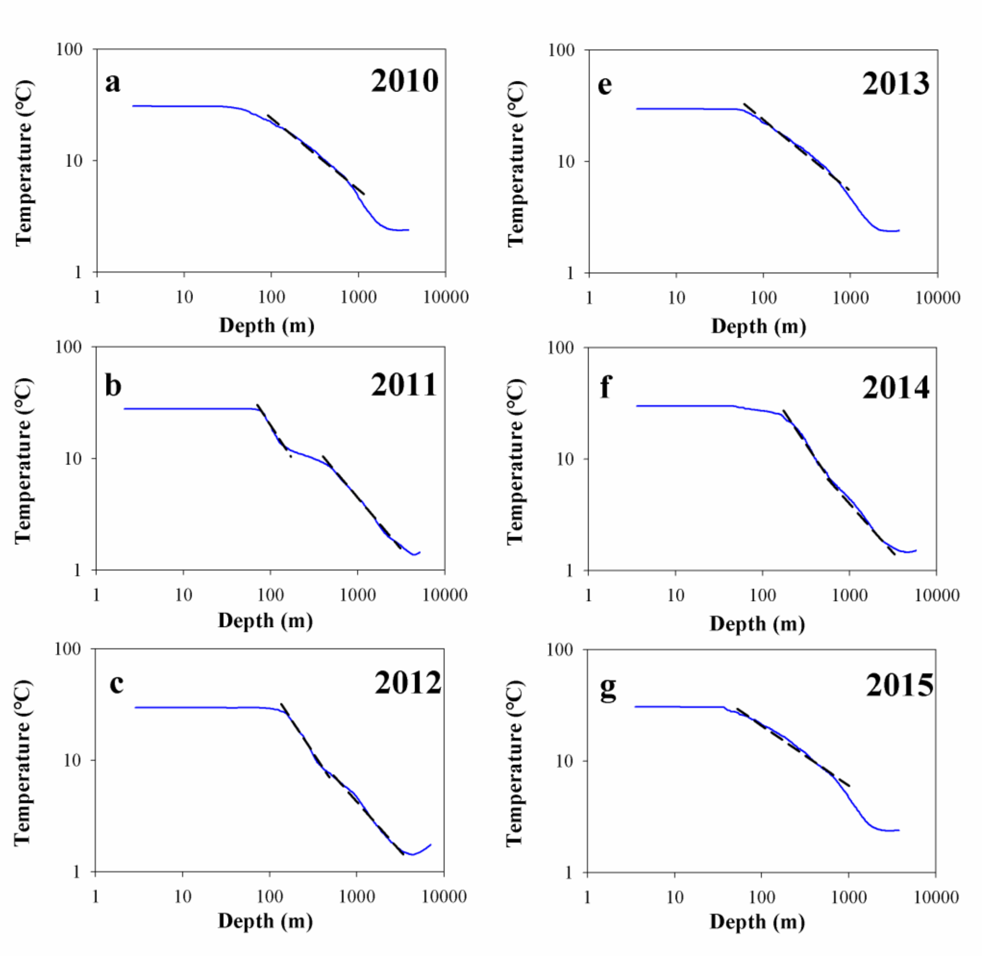

Figure S2. Log–log plot of the relationship between temperature and water depth at five representative locations. The short dash segment is the fitting line. The coefficients of determination (R^2) values for the short dash segments range from 0.91 to 0.99.

Table S1. Correlation analysis between temperature and conductivity.

Year	Control Variables			Conductivity
2010	None	Temperature	Correlation	0.995
			Significance (2-tailed)	.000
			df	1234
	Pressure and Salinity	Temperature	Correlation	0.999
			Significance (2-tailed)	.000
			df	1232
2011	None	Temperature	Correlation	0.985
			Significance (2-tailed)	.000
			df	1928
	Pressure and Salinity	Temperature	Correlation	0.999
			Significance (2-tailed)	.000
			df	1926
2012	None	Temperature	Correlation	0.980
			Significance (2-tailed)	.000
			df	2311
	Pressure and Salinity	Temperature	Correlation	0.999
			Significance (2-tailed)	.000
			df	2309
2013	None	Temperature	Correlation	0.997
			Significance (2-tailed)	.000
			df	3611
	Pressure and Salinity	Temperature	Correlation	0.999
			Significance (2-tailed)	.000
			df	3609
2014	None	Temperature	Correlation	0.989
			Significance (2-tailed)	.000
			df	5751
	Pressure and Salinity	Temperature	Correlation	0.999
			Significance (2-tailed)	.000
			df	5749
2015	None	Temperature	Correlation	0.996
			Significance (2-tailed)	.000

		df	3752
Pressure and Salinity	Temperature	Correlation	0.999
		Significance (2-tailed)	.000
		df	3750

Table S2. Detailed information of three CTD profiles in the Atlantic.

Site	Latitude (E)	Longitude (N)	NODC Cruise ID	Year	Month
a	47	-40.005	US-35482	2011	May
b	-15.4993	-24.9998	US-35879	2014	January
c	24.4985	-25.35729	GB-13253	2016	January
